# Tolerability and effects on quality of life of liposomal nasal spray treatment compared to nasal ointment containing dexpanthenol or isotonic NaCl spray in patients with rhinitis sicca

**DOI:** 10.1007/s00405-013-2362-y

**Published:** 2013-01-31

**Authors:** C. Hahn, M. Böhm, S. Allekotte, R. Mösges

**Affiliations:** Institute of Medical Statistics, Informatics and Epidemiology (IMSIE), University of Cologne, 50924 Cologne, Germany

**Keywords:** Dry nose, Rhinitis sicca, Liposomes, Dexpanthenol, NaCl spray, Quality of life

## Abstract

This study aimed to investigate symptom reduction via the liposomal nasal spray LipoNasal (LN) in patients with rhinitis sicca. Tolerability and the impact on quality of life were also examined. The same parameters were established in parallel for treatment approaches with Bepanthen (BP) nasal ointment containing dexpanthenol and the Rhinomer (RH) nasal spray containing NaCl. This prospective, controlled, open-label observation study was a multicenter trial. 92 patients with rhinitis sicca were allocated to three arms according to their symptoms: LN: *n* = 33; BP: *n* = 32 and RH: *n* = 27. The study comprised three visits at an interval of 14 days. Efficacy was examined by the Rhinitis Sicca Symptom Score (RSSS) documented daily and at the visits based on an endoscopic evaluation. The nasal spray sensory scale was used to investigate the tolerability. Quality of life (QoL) was measured by means of the Rhinoconjunctivitis Quality of Life Questionnaire (RQLQ) and the “Short Form 12” of the “Impact on Health-Related Quality of Life (HRQL)” questionnaire on general quality of life. Nasal symptoms improved significantly (*p* = 0.001) under all three treatment approaches, reflected by the reduction in the RSSS and the Endoscopy Sum Score. A comparison of the three groups showed that no therapy was significantly superior to any of the others (*p* = 0.410). The tolerability of all treatments was good. Concerning the nasal moisturization, LipoNasal was evaluated better than Bepanthen and Rhinomer. Quality of life improved in all groups, but not significantly. The results show good efficacy and tolerability of the liposomal nasal spray compared to generally recognized treatments of rhinitis sicca with dexpanthenol nasal ointment and NaCl nasal spray. LipoNasal therefore constitutes a good treatment for patients suffering from dry nose.

## Introduction

The term “dry nose” has not yet been uniformly defined [1]. Otolaryngologists often use the terms “rhinitis sicca” or “dry rhinitis,” although no clear definition exists; characteristic for the disorder, however, is hypotrophy of the nasal mucosa [2].

The symptoms of rhinitis sicca are manifold and range from the subjective sensation of a dry nose to visible crusting. Because of the dry nasal mucosa, patients often suffer from various combinations of rhinitis sicca symptoms: sensation of dryness in the nose, itching, mild burning, impaired nasal breathing, crusting, possibly with unpleasant odor (ozena), epistaxis, anosmia, and concomitant pharyngitis.

The mechanical and functional intactness of the mucus membranes is an important defense mechanism against infections. Depending on how much they dry out, mucociliary transport or even the epithelial barrier can be adversely affected. Such symptoms can markedly impair the quality of life of affected patients and result in considerable socio-economic burdens, especially in the case of fetid crusting in so-called ozena as the severest form of rhinitis sicca. These patients therefore visit ears–nose–throat (ENT) offices often and repeatedly.

Used for treatment of rhinitis sicca anterior and after nasal and sinus surgery, dexpanthenol has been available as a nasal ointment without preservatives (Bepanthen^®^ Roche Eye and Nose Ointment) since the 1960s [3]. Dexpanthenol is an active ingredient that promotes wound healing. It is converted in vivo to pantothenic acid, a component of coenzyme A which in turn activates fibroblast proliferation and accelerates the reepithelialization process [4]. In a study by Kehrl and Sonnemann [5], the effect of dexpanthenol in rhinitis sicca has been scientifically verified.

Other local treatment approaches consist of nasal rinses, moisturizing nasal sprays (NaCl), inhalations, and oils. Nasal rinses are listed in the guidelines for treating rhinosinusitis, although they are not explicitly recommended for use in rhinitis sicca [6]. The positive effect of nasal rinses and nasal sprays containing NaCl is based on moistening the nasal mucosa and softening of any existing crusts.

An alternative to local therapy of dry nose is the application of LipoNasal^®^ nasal spray, which contains liposomes composed of phospholipids, fatty acids, and vitamin E. This therapy approach involves the supplementation of phospholipids as the most important component of the body’s own surfactant. It is meant to restore the protective secretion film, whose functions are moistening, mucosal defense, and mucociliary transport [7, 8].

The study described here investigated the properties of this liposomal nasal spray in the treatment of patients suffering from rhinitis sicca in routine clinical practice. Data on the efficacy, tolerability, and quality of life were gathered to this purpose. The investigation was designed as a prospective, nonrandomized observation study with an active control. The comparative groups were treated with a dexpanthenol nasal ointment and a nasal spray containing NaCl. The present study was conducted in compliance with good clinical practice guidelines. Since the nasal sprays and ointment are available without a prescription, approval by an ethics committee was not required. Prior to the study, however, a professional legal consultation took place with the appropriate ethics committee.

## Methods

### Study design

This trial was a prospective, controlled, open-label observation study. From 12 May 2011 to 12 December 2011, a total of 92 patients were enrolled at seven ENT trial sites. All patients were 12 years or older and suffered from rhinitis sicca. Patients visited the trial site because of symptoms resulting from this condition. Restricted inclusion of patients in the post-marketing surveillance study based on the indication of rhinitis sicca and strict adherence to the principle of non-intervention allowed data to be collected for the most unselective patient population as possible. The investigators, taking the patient into consideration, were free to decide who was to receive which medication. The study consisted of a total of 28 ± 2 observation days, where Visit 1 took place on the first day (V1), Visit 2 after 14 ± 2 days (V2), and Visit 3 after 28 ± 2 days (V3).

### Medication

LipoNasal^®^ Nasenpflege Spray (LN) is produced by Optima Pharmazeutische GmbH, Moosburg. Other ingredients of the nasal spray than the liposomes are soja lecithin, sodium chloride, ethanol, dexpanthenol, vitamin A palmitate, vitamin E, and aqua purificata. Treatment was conducted according to the information in the package leaflet. On average, 3–3½ sprays of LN per nostril per day were used; in the course of a day, a total of 0.5–0.6 ml of the liposomal suspension was applied to the nasal mucosa.

Bepanthen^®^ Eye and Nose Ointment (BP), manufactured by Bayer Vital GmbH, Leverkusen, is a product that promotes wound healing. Dexpanthenol is the active ingredient (1 g ointment contains 0.05 g dexpanthenol). Other components are: rac-(3R)-3-hydroxy-4.4-dimethyloxolan-2-one; lanolin; viscous paraffin; petroleum jelly; water for injection. A 1-cm long ribbon of ointment should be applied to the nasal mucosa and rubbed in gently once to several times daily. The patients used an average of 2–2½ ribbons per nostril per day.

Rhinomer^®^ Nasal Spray (RH) is a medicinal product made by Novartis Consumer Health GmbH, Munich. RH contains sterile, isotonized seawater spray. When applied in rhinitis, it serves to moisten the nasal mucosa and supports the cleansing function of the mucosa’s ciliated epithelium. A dose of 1–2 sprays in each nostril several times a day as needed is recommended. RH was used between 2½ and 3 times daily on average.

### Study protocol

Demographic data were recorded during an admission interview and physical examination on Visit 1 by ENT specialists.

The documented number of sprays applied per nostril per day allowed a better understanding of the amount of nasal spray used and patient compliance.

Efficacy was examined via the Rhinitis Sicca Symptom Score (RSSS). The severities of the symptoms dryness, obstruction of nasal breathing, and crusting were rated on an ordinal scale of 0–3 (0 = no, 1 = mild, 2 = moderate, 3 = severe), and the individual values were added to obtain a sum score. In addition, the occurrence of frequent concomitant symptoms such as itching and pain in the nose, concomitant pharyngitis, epistaxis, and anosmia were documented. These symptoms were recorded at each visit by the investigator, but also daily by the patient himself in a diary. In order to make an additional objective assessment of efficacy possible, the investigator performed an endoscopic examination of the nasal cavity at Visits 1, 2, and 3 and classified crusting, dryness of the mucosa, redness and swelling of the inferior and middle nasal turbinates, and possible ulcerous changes of the nasal mucosa on a 3-point scale (0 = no, 1 = mild, 2 = severe). The endoscopy score (ES) was calculated from these data.

After applying the respective nasal spray for the first time, the patient specified the perceived duration of action (only for minutes, <1, 1–2, 2–4, >4 h, no effect).

The Nasal Spray Sensory Scale (NSSS) served to examine tolerability [9] by measuring the patients’ sensory perception immediately following the first nasal spray application and 2 min thereafter. Fourteen questions pertaining to sensory parameters could be answered by marking a visual analog scale (0 = poor evaluation, 100 = good evaluation). The safety of the treatments was examined via exact documentation of adverse events.

Since no disease-specific quality of life questionnaire exists designed especially for patients with rhinitis sicca, the Rhinoconjunctivitis Quality of Life Questionnaire (RQLQ) [10] was used which was developed for rhinoconjunctivitis patients. The SF-12 [11] questionnaire was also implemented to gather general quality of life data.

Before and after treatment, patients were asked to assess the impairment intensity of every item on a point scale; changes in quality of life could thus be evaluated.

Patients also assessed their subjective well-being daily on a visual analog scale (0 = very poor, 100 = very good).

At the end of the observation period, the investigator and patients were able to make a final positive or negative evaluation of efficacy and tolerability of the nasal spray used.

### Statistical methods

Data were evaluated using SPSS 18 statistics software by SPSS Inc. Patient data were entered in the SPSS database two times each by two independent people, and a check for errors was made thereafter. Input errors were corrected.

At first, all data were analyzed descriptively. After testing for normal distribution using the Kolmogorov–Smirnov test, differences of non-normally distributed dependent variables were examined for significance by means of the Wilcoxon test and independent variables via the Mann–Whitney *U* test. The significance level was set at α = 0.05 %. Missing values were generally treated as “missing values”.

## Results

### Homogeneity of treated groups at baseline

In all, 92 patients were enrolled in the post-marketing surveillance study, 32 patients of which were included in the LN treatment group, 32 patients in the BP group, and 27 patients in the RH group.

Treatment group LN consisted of 19 female and 14 male patients. 20 female and 12 male patients were included in the BP treatment group, and 14 female and 13 male patients were enrolled in the RH group.

Patient ages ranged from 19 to 88 years, the average age being 56 ± 17.3 years. Age distribution was similar in the three groups and averaged 51 ± 16.1 years in the LN group, 58 ± 18.5 years in the BP group, and 60 ± 17.3 years in the RH group (Table 1).

**Table 1:** Demographic data

	LN	BP	RH
Number of patients	33	32	27
Sex (male/female)	14/19	12/20	13/14
Mean age (years)	51 ± 16.1	58 ± 18.5	60 ± 17.3

Patients in the LN group had suffered for about 3½ years on average from rhinitis sicca, patients in the BP group for an average of almost 9 years, and patients in the RH group for an average of 6½ years.

### Efficacy

Like both of the comparative therapies, LN treatment resulted in significant improvement in the RSSS (*p* < 0.001) and the ES (*p* = 0.001) from V1 to V3 that had been assessed by specialists (Table 2). It was a similar situation for the RSSS documented daily by the patients in a diary.

**Table 2:** Efficacy of symptom reduction (Wilcoxon Test)

	LN		BP		RH	
	Symptom score	*p* values	Symptom score	*p* values	Symptom score	*p* values
RSSS Visit 1	5.39 ± 1.999	<0.001	5.81 ± 2.151	<0.001	4.62 ± 2.099	<0.001
RSSS Visit 2	3.60 ± 1.754		3.00 ± 1.944		3.39 ± 2.190	
RSSS Visit 3	2.83 ± 2.037		2.64 ± 2.129		2.10 ± 0.995	
ES Visit 1	4.45 ± 1.697	<0.001	4.48 ± 1.671	<0.001	3.98 ± 1.385	<0.001
ES Visit 2	2.77 ± 1.455		2.57 ± 1.476		2.87 ± 1.842	
ES Visit 3	2.55 ± 1.682		2.11 ± 1.672		2.33 ± 1.528	

Considering the occurrence of frequent concomitant symptoms the symptom of itching improved to different degrees in all three treatment groups: LN from 46.9 to 39.3 %, BP from 50.0 to 35.7 %, and RH from 57.7 to 25.0 %, which constitutes the best result.

At the start of treatment, 42.4 % of patients in the LN group suffered from concomitant pharyngitis. In comparison to the treatment groups BP (161 %) and RH (15.4 %), the number of affected patients is higher. After 4 weeks, only 21.4 % of patients in the LN group, no one in the BP group, and 5 % of patients in the RH group said they had suffered from concomitant pharyngitis.

Impaired olfaction could be improved only minimally in the LN group (from 66.7 to 53.6 %). In the BP group, it could be reduced from 62.5 to 28.6 % and in the RH group from 53.8 to 35.0 %.

Pain in the nose diminished considerably during the course of treatment in all three groups. In the LN group it improved from 28.1 at V1 to 10.7 % at V3, in the RN group from 26.9 to 10.0 % and in the Bepanthen group, pain disappeared almost completely (from 31.3 to 3.6 %).

The situation was similar for nosebleeds. About one-third of the patients suffered from them at study inclusion. In all three groups, this symptom almost completely disappeared after 4 weeks of treatment (in the LN and BP groups, one patient each was affected by this symptom). Table 3 shows the number of patients affected at baseline and after 4 weeks.

**Table 3:** Occurance of concomitant symptoms

Patients affected	LN		BP		RH	
	*n* = baseline	*n* = 4 weeks	*n* = baseline	*n* = 4 weeks	*n* = baseline	*n* = 4 weeks
Concomitant pharyngitis	14	6	5	0	4	1
Pain	9	3	10	1	7	2
Nosebleeds	10	1	8	1	8	0
Impaired olfaction	22	15	20	8	14	7

### Duration of action

The evaluations of the three trial medications produce an inconsistent picture when compared. 27 % of the patients in the RH group stated that they had felt no effect of the nasal spray after applying it. In group BP, only 10 % of the patients made the same assessment, and only about 5 % of patients in the LN group indicated the same. 26 % of the patients who received LN specified a duration of action of “1–2 h,” while it was approximately 18 % in the BP and RH groups.

In the LN and BP groups, ca. 35 % of all patients indicated a duration of action of “2–4 h”, whereas it was only about 25 % in the RH group. “over 4 h” duration of action was most often stated by the patients in the BP group at approximately 30 %; about 21 % in the LN group and approximately 15 % in the RH group indicated the same effect.

**Fig. 1:**
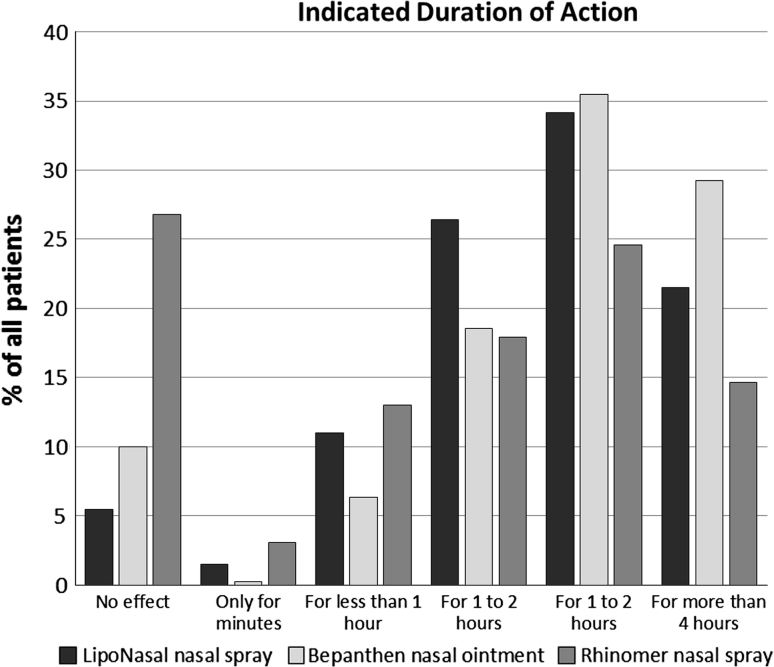
Comparison of the duration of action within the three groups

One thus recognizes the clearly poorer assessment of duration of action for the test medication Rhinomer in comparison to the other two products. Bepanthen was generally evaluated as having the longest duration of action (Fig. 1).

### Tolerability and safety

When analyzing the Nasal Spray Sensory Scale, differences were observed among the three types of treatment (Table 4). BP and RH achieved better results than LN, especially in terms of smell and taste. In assessing nasal moisturization, however, LN was evaluated better (LN 71.75 ± 22.277, BP 60.28 ± 24.454, and RH 67.08 ± 23.202 out of a possible 100 points).

**Table 4:** Four of the 14 items of the nasal spray sensory scale

	LN	BP	RH
Odor intensity Visit 1	68.42 ± 26.690	82.06 ± 16.240	87.00 ± 21.195
Odor intensity Visit 2	79.10 ± 22.768	80.07 ± 18.360	82.65 ± 22.793
Odor intensity Visit 3	74.67 ± 19.944	85.67 ± 12.551	84.45 ± 21.387
Intensity of taste Visit 1	76.45 ± 24.258	88.61 ± 16.814	87.81 ± 19.468
Intensity of taste Visit 2	63.69 ± 27.909	85.36 ± 20.447	84.70 ± 18.605
Intensity of taste Visit 3	72.07 ± 19.036	85.07 ± 22.072	87.68 ± 13.375
Nasal moisturization Visit 1	71.75 ± 22.277	60.28 ± 24.454	67.08 ± 23.202
Nasal moisturization Visit 2	66.28 ± 24.721	62.36 ± 24.497	67.73 ± 21.472
Nasal moisturization Visit 3	70.59 ± 19.753	68.07 ± 21.958	74.90 ± 19.620
Overall impression Visit 1	59.25 ± 28.098	71.47 ± 22.446	74.37 ± 19.960
Overall impression Visit 2	68.31 ± 21.032	69.39 ± 25.300	81.00 ± 15.814
Overall impression Visit 3	65.15 ± 18.749	80.46 ± 17.648	76.30 ± 21.379

To be able to recognize a possible habituation to the nasal treatment, the patients were also asked to evaluate their nasal spray/ointment at Visits 2 and 3. It became apparent that the patients perceived the odor intensity of LipoNasal (74.67 ± 19.944) at Visit 3 somewhat more pleasant than at V1 (68.42 ± 26.690).

Adverse events occurred during the treatment phase in all three treatment groups (3 in LN, 3 in BP, and 1 in RH).

Under treatment with LN, one patient with pre-existing bronchial asthma claimed to have suffered dyspnea 10 days after the start of treatment. This feeling occurred regularly and was moderately strong. After discontinuation of the trial medication, dyspnea disappeared, which does not rule out a correlation with the trial medication.

Furthermore, one case of acute rhinitis and one disorder not further described occurred, both resulting in early termination of the study.

Adverse events in the BP group were mild pharyngitis, olfactory problems under lasting treatment with BP nasal ointment, increased encrusting, and headache occurring in one female patient. A correlation to the trial medication cannot be ruled out.

In the RH group, increasing impairment of nasal breathing and moderate headaches occurred occasionally/periodically in one patient. Symptoms resolved after adapting concomitant therapy; here, too, a connection to the trial medication cannot be ruled out.

### Quality of life

**Fig. 2:**
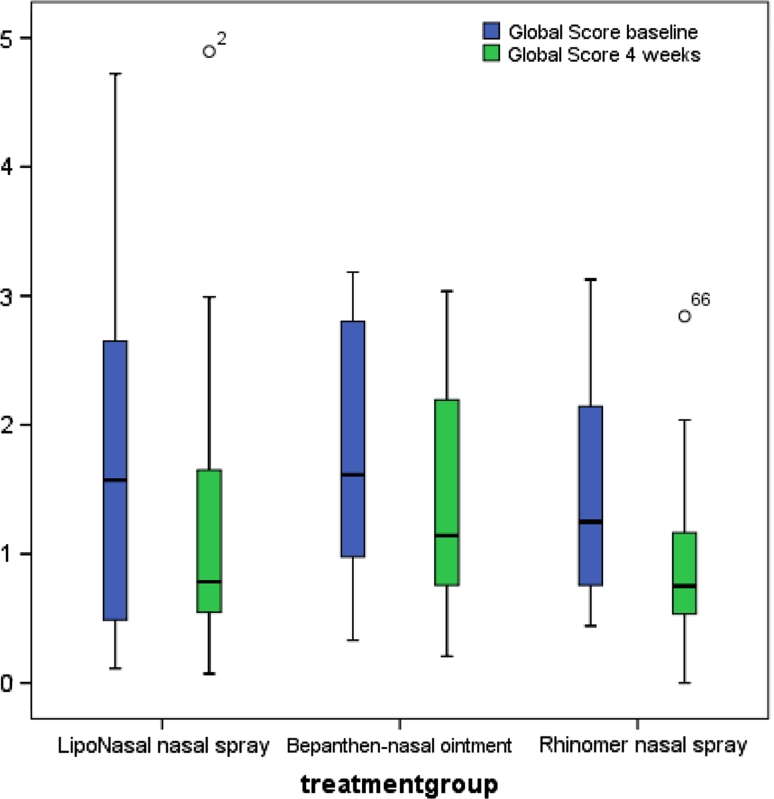
Development of RQLQ global score

The RQLQ global score for quality of life declined under LN therapy from a rating of “somewhat restricted” at V1 to “few restrictions” at V3. Fewer health restrictions were also stated in the BP and RH group. In the RH group the baseline value was somewhat lower than the values for LN and BP (Fig. 2).

The results show that significant improvement in the RQLQ global score could be achieved with all three trial medications between V1 and V3 (*p* ≤ 0.05).

In the category “impairment of sleep,” only LN was able to achieve significant improvement from V1 to V3 (*p* = 0.003). When observing the number of valid values at V1 and V3, respectively; however, it appears that the statistical significance is only achieved due to study dropouts.

The Kruskall–Wallis test was able to show that no significant difference existed in the improvement of the sum score among the three groups (*p* > 0.05).

The analysis of the SF-12 questionnaire also showed a tendency toward improvement in the sum scales for physical and mental health. The higher the values, the better the patients assessed their quality of life. For physical health, values in the LN group rose from 45.05 ± 11.736 to 48.64 ± 8.518 and in the BP group from 45.84 ± 7.937 to 48.12 ± 7.498. In the RH group, the baseline value was somewhat higher at 49.37 ± 7.596, but did not improve by V3.

In terms of mental health, however, the values of the RH group improved more strongly from 48.93 ± 9.620 to 53.16 ± 8.198. In the LN group, the values rose from 48.65 ± 9.260 to only 49.00 ± 10.898, and in the BP group from 47.99 ± 9.470 to 49.80 ± 10.182.

The Wilcoxon test, however, showed that these improvements in physical and mental health from V1 to V3 were not significant in any group (*p* > 0.05). The Kruskall–Wallis test showed that here, too, there is no significant difference among the three groups (*p* > 0.05).

As for the patient’s subjective state of health, all groups had baseline values between 50 and 60 documented in their patient diaries, with the values reaching about 70 points near the end of treatment. The higher the values, the better the patient assessed his or her subjective state of health. This may indicate that all three treatment forms had a positive effect on the patients’ subjective state of health.

### Final assessment

The efficacy of the three trial medications was assessed somewhat differently by the investigators and the patients. About two-thirds of the investigators described the efficacy of all three medications as “good” or “very good.”

It appears that in contrast to the investigators, patients tended to evaluated efficacy more poorly. In the LN group, half of the patients rated efficacy more negatively; in the BP group, it was somewhat less at 40 % of patients and in the RH group over 60 % assessed efficacy more negatively. Nevertheless, 73.9 % of the patients would recommend LN nasal spray, 75 % BP, and over 80 % RH.

Generally, it can be said that patients rated the efficacy of BP the best, followed by LN and RH, which received the poorest ratings.

## Discussion

The aim of this observational study was to gain insight into the tolerability and the effects on the quality of life of a liposomal nasal spray, a nasal ointment containing dexpanthenol, or an isotonic NaCl spray in patients with rhinitis sicca under practical conditions. Also to be investigated was the extent to which the treatment influenced the condition of the nasal mucosa upon endoscopic evaluation and the severity of the patients’ symptoms.

A total of 92 patients with a wide variety of disease severities participated in this post-marketing surveillance study.

### Efficacy

Four-week treatment of the three patient groups led to a significant reduction in the RSSS (LN 48 %, BP and RH 55 %) and to an improvement in the ES (LN 43 %, BP 53 %, and RH 41 %) for all three investigational products compared to the assessment prior to treatment start (*p* < 0.001). As early as after 2 weeks of treatment, symptoms decreased significantly. When comparing the three test products, no superiority or inferiority could be ascertained.

The improvements achieved in RSSS and ES show a therapy effect for the liposomal nasal spray as well as an effect for both comparative treatments with dexpanthenol nasal ointment and isotonic NaCl spray, respectively.

In the study by Kehrl and Sonnemann [5] on the treatment of rhinitis sicca anterior with dexpanthenol nasal spray (active group *n* = 24) or isotonic saline solution (placebo group *n* = 24), nasal airway obstruction and the extent of crust formation were also assessed to evaluate efficacy (our study also rated dryness of the nose). The results for the dexpanthenol product were comparable to our results: significant improvements in nasal airway obstruction after 2 and 4 weeks of treatment. In the group using the isotonic NaCl spray, however, no significant improvements were achieved, which was just the opposite case in our study.

Verse et al. [12] compared dexpanthenol nasal ointment with dexpanthenol nasal spray based on mucociliary clearing time. This test was not performed by any investigator in our study due to time constraints under practical conditions; therefore, it is not possible to draw a comparison here.

Müller-Sacks [13] was able to achieve similarly good results in his study with nasal sprays containing NaCl. A post-marketing surveillance study using NaCl nasal spray was conducted with 205 airline employees who suffered from rhinitis sicca symptoms at least occasionally. In 88.8 % of the participants, a very good or good moisturization of the nasal mucosa was attained through application of the spray, and in more than half of the patients crusting decreased. Overall, 89 % of participants confirmed symptom improvement, which in 63.7 % of cases also meant an increase in quality of life.

In earlier studies with the liposomal nasal spray in patients with seasonal allergic rhinitis, a significant reduction in nasal (*p* = 0.003) and conjunctivitis (*p* = 0.005) symptoms could be achieved and quality of life improved (LN: *p* = 0.002) [14–16]. Attention was thereby drawn to the possible therapeutic potential in patients with rhinitis sicca.

The liposomal nasal spray investigated here for the first time for application in rhinitis sicca thus lessens the symptoms of dry nose to approximately the same extent than the comparative products already proven in several studies to be effective.

### Duration of action

In the present post-marketing surveillance study, the duration of action of the liposomal nasal spray was evaluated better than that of the isotonic NaCl spray, although the dexpanthenol nasal ointment was rated best of all three medicinal products. No information can be found in the literature with regard to the duration of action of the individual products.

### Tolerability and safety

The tolerability of all three preparations can be evaluated as good. The analysis of the NSSS at V1 showed only slight differences in the assessment of the three treatment approaches. Worth noting is that LN performed better on average in moisturizing the nose than the other two preparations (LN 71.75 ± 22.277, BP 60.28 ± 24.454, RH 67.08 ± 23.202). The high standard deviation values make clear how differently the patients rated their treatments. For the rest of the parameters, LN nasal spray performed slightly worse than BP and RH, especially for the parameters smell and taste. Similar results have already been observed in other studies on LN [15]. Based on these observations, a new formula for optimizing smell and test has been used. The new LN was rated considerably better than the old formula, but it does not quite achieve the same values as the comparative preparations in the assessment of tolerability.

The only adverse event for LN described in detail was dyspnea in one asthma patient. Such an effect has not been observed in any of the previously conducted studies with LN [14–16] and was caused in all likelihood by the hyperreactive bronchial system, typical in asthma patients and irrespective of application of the specific product.

In two patients from the BP group and one patient from the RH group, exacerbation of existing symptoms, such as olfactory disorders, crusting, and nasal obstruction, has been described. The question remains open here, too, as to how these symptoms could have been aggravated. Possible intolerances cannot be ruled out.

### Quality of life

Significant improvement in the RQLQ global score between V1 and V3 could be achieved with all three test medications (*p* ≤ 0.05). No significant difference, however, existed among the three groups (*p* > 0.05).

In the category “sleep problems,” only LN could achieve significant improvement from V1 to V3 (*p* = 0.003). Therefore, it is clear that relatively insignificantly appearing diseases also have an effect on quality of life. It is questionable whether it is also clinically relevant.

In recording health-related quality of life via the SF-12 questionnaire, values tended to be better after 4 weeks of treatment; however, these values were not statistically significant.

### Final evaluation

The varying final assessments made by the patients with respect to efficacy are not plausible at first glance, since significant symptom reductions took place in all three groups and no one therapy was superior to any other. It may possibly be explained in light of the better taste of the nasal ointment and NaCl nasal spray.

## Conclusion

Treatment of rhinitis sicca with LipoNasal nasal spray is a therapy form well accepted by patients and has a positive safety profile for side effects.

It is in no way inferior to the other two comparative treatments in terms of efficacy and its positive effect on quality of life. In particular due to the best assessment with respect to moisturizing, LipoNasal can be assessed as a good treatment for patients suffering from dry nose.
